# *LINC01133* Inhibits Invasion and Promotes Proliferation in an Endometriosis Epithelial Cell Line

**DOI:** 10.3390/ijms22168385

**Published:** 2021-08-04

**Authors:** Iveta Yotova, Quanah J. Hudson, Florian M. Pauler, Katharina Proestling, Isabella Haslinger, Lorenz Kuessel, Alexandra Perricos, Heinrich Husslein, René Wenzl

**Affiliations:** 1 Department of Obstetrics and Gynecology, Medical University of Vienna, Waehringer Guertel 18-20, A-1090 Vienna, Austria; quanah.hudson@univie.ac.at (Q.J.H.); katharina.proestling@meduniwien.ac.at (K.P.); isabella.haslinger@meduniwien.ac.at (I.H.); lorenz.kuessel@meduniwien.ac.at (L.K.); alexandra.perricos@meduniwien.ac.at (A.P.); heinrich.husslein@meduniwien.ac.at (H.H.); 2 Institute of Science and Technology Austria, Am Campus 1, 3400 Klosterneuburg, Austria; florian.pauler@ist.ac.at

**Keywords:** endometriosis, long noncoding RNAs, lncRNAs, epithelial to mesenchymal transition, EMT, proliferation, migration, cytoskeleton

## Abstract

Endometriosis is a common gynecological disorder characterized by ectopic growth of endometrium outside the uterus and is associated with chronic pain and infertility. We investigated the role of the long intergenic noncoding RNA 01133 (*LINC01133*) in endometriosis, an lncRNA that has been implicated in several types of cancer. We found that *LINC01133* is upregulated in ectopic endometriotic lesions. As expression appeared higher in the epithelial endometrial layer, we performed a siRNA knockdown of *LINC01133* in an endometriosis epithelial cell line. Phenotypic assays indicated that *LINC01133* may promote proliferation and suppress cellular migration, and affect the cytoskeleton and morphology of the cells. Gene ontology analysis of differentially expressed genes indicated that cell proliferation and migration pathways were affected in line with the observed phenotype. We validated upregulation of p21 and downregulation of Cyclin A at the protein level, which together with the quantification of the DNA content using fluorescence-activated cell sorting (FACS) analysis indicated that the observed effects on cellular proliferation may be due to changes in cell cycle. Further, we found testis-specific protein kinase 1 (TESK1) kinase upregulation corresponding with phosphorylation and inactivation of actin severing protein Cofilin, which could explain changes in the cytoskeleton and cellular migration. These results indicate that endometriosis is associated with *LINC01133* upregulation, which may affect pathogenesis via the cellular proliferation and migration pathways.

## 1. Introduction

Endometriosis is a disorder characterized by the presence of endometrial tissue outside of the uterine cavity, most often attached to organs of the peritoneal cavity [1]. As a common gynecological disorder affecting 6–10% of reproductive age women, endometriosis presents a significant burden on affected patients and society. However, the pathogenesis of the disease is still not well defined. The most accepted explanation for the origin of the cells from which endometriosis lesions develop is retrograde menstruation, whereby endometrial cells flow out into the peritoneal cavity via the fallopian tubes [1]. In order to establish a lesion, endometrial cells in the peritoneal cavity must adhere, implant, and differentiate while avoiding the immune system. Thus, complex interactions between molecular, humoral, immune, genetic and epigenetic signals must occur to support the development, growth and persistence of a lesion [2].

The advent of next-generation sequencing has accelerated identification of changes in the transcriptome, genome and epigenome in the pathogenesis of human diseases including endometriosis. In recent years, it has become clear that interactions between protein-coding (mRNA) and non-coding transcripts such as long-non-coding RNAs (LncRNAs) can influence the development of disease.

LncRNAs are a class of RNAs greater than 200 nucleotides in length that show similar RNA biology features to mRNAs but do not code for a protein [3]. LncRNA can be transcribed from different genomic regions, including introns, exons, and intergenic regions. Around 30,000 lncRNAs have been identified in humans and mice [4], but only a fraction of these have so far been demonstrated to be functional. LncRNAs are less evolutionally conserved than mRNA [5], and are thought to form a complex tertiary structure when binding DNA, RNA and proteins that may be required for their function [6]. They may act as epigenetic gene regulators by affecting biological functions in the cell such as the assembly and function of nuclear bodies, the stability and translation of cytoplasmic mRNAs, and signaling pathways [7,8]. LncRNAs have been reported to regulate gene expression in a number of different ways, including targeting chromatin modifiers such as Polycomb repressive complex 2 (PRC2), or by acting as so-called miRNA sponges to bind miRNAs that would otherwise bind other targets thereby affecting their expression [9]. Several studies using patient samples and animal models have reported aberrant expression of long non-coding RNAs in endometriosis [10,11]. A growing body of evidence has identified lncRNAs that can alter cell proliferation, migration, invasion and apoptosis of endometriosis cells [12,13,14]. The molecular mechanism by which these lncRNAs cause these phenotypes has not been shown in all cases. LncRNAs have also been associated with endometriosis-associated angiogenesis [15], infertility [12] and epithelial to mesenchymal transition (EMT) [16]. EMT is a cellular process where epithelial cells acquire a more invasive mesenchymal phenotype and is associated with the loss of E-cadherin (CDH1) and a gain of N-cadherin (CDH2), and the presence of EMT promoting factors such as TWIST1, SNAIL, SLUG and TGFβ [17,18]. In a pathogenic context, it has been established that EMT is a key process in carcinogenesis, but also plays a less well-characterized role in endometriosis lesion development [19]. Hence, clarifying the role of EMT in endometriosis, and its regulation by pathways that may include lncRNAs remains an important issue in the field.

*LINC01133* is an lncRNA that has recently been identified as a putative prognostic marker for endometrial cancer [20]. It has also been associated with the regulation of EMT in several cancers including cervical [21], breast [22], colorectal [23] and gastric [24]. Given that EMT is also a feature of endometriosis [25] we reasoned that *LINC01133* may also be involved in the pathogenesis of endometriosis, and sought to investigate this in our study.

## 2. Results

### 2.1. LINC01133 Is Upregulated in Ectopic Endometriosis Lesions

**Figure 1:**
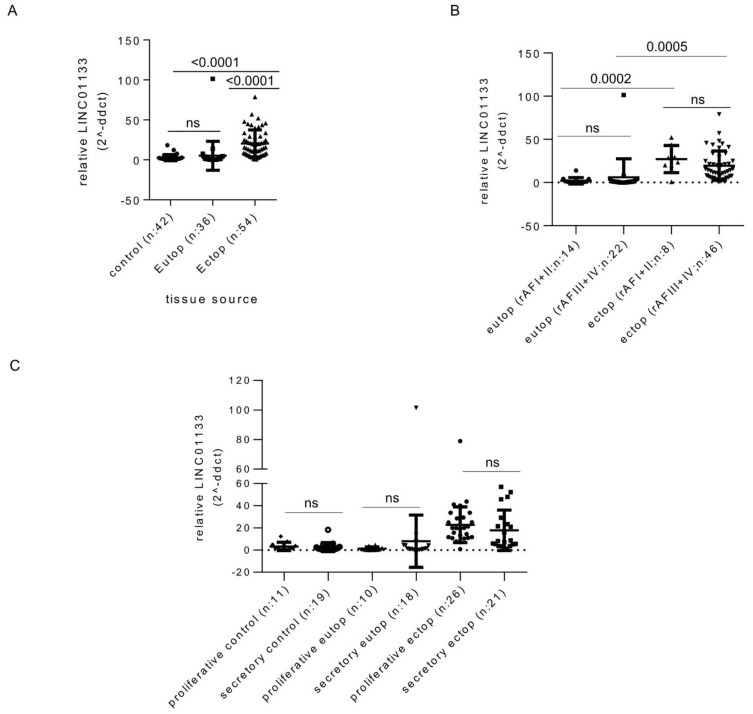
*LINC01133* expression is upregulated in endometriosis lesions. (**A**) Relative expression of *LINC01133* is significantly increased in ectopic endometriotic lesions compared to the eutopic endometrium of women with and without endometriosis. Expression in the eutopic endometrium does not differ between women with and without the disease. (**B**) In endometriosis patients, *LINC01133* expression does not significantly differ between mild (rAF I + II) and more severe (rAF III + IV) disease stages, in either the eutopic endometrium or ectopic lesions. (**C**) *LINC01133* expression does not significantly differ between the proliferative and secretory stages of the menstrual cycle in control eutopic endometrium, eutopic endometrium from patients, or ectopic lesions. Data in (**A**–**C**) are presented as dot plots including the mean relative expression levels and standard deviation in each group As the sample sizes were not equal, data were analyzed by fitting a mixed model, rather than repeated measures ANOVA (which requires equal sample size). Adjusted *p*-values values (adjp) < 0.05 were considered significant with non-significant differences indicated by ns. Control: endometrial tissue of women without endometriosis, Eutop: endometrial tissue of women with endometriosis, Ectop: endometriosis lesions.

In order to identify changes that may occur in *LINC01133* expression levels during endometriosis pathogenesis, we compared control eutopic endometrium from women without endometriosis with eutopic endometrium from endometriosis patients, and ectopic endometriosis lesions using quantitative reverse transcription PCR (qRT-PCR). This showed that *LINC01133* expression is significantly upregulated in endometriosis lesions compared to eutopic tissue of both patients and controls (Figure 1A). These changes were independent of disease stage and menstrual cycle phase (Figure 1B,C). We next used RNA Scope in situ hybridization to determine *LINC01133* spatial localization within endometrium tissue. We found that *LINC01133* is expressed in both stromal and epithelial cells of the eutopic endometrium of women with endometriosis, but appeared to show higher levels in glandular epithelial cells (Figure S1A). Quantification confirmed this observation, with positive glandular epithelial cells around five times more frequent than positive stroma cells (*p* = 0.0012) (Figure S1B).

### 2.2. LINC01133 siRNA Knockdown in 12Z Endometriosis Epithelial Cells Leads to Transcriptional Deregulation of 1210 Genes

**Figure 2:**
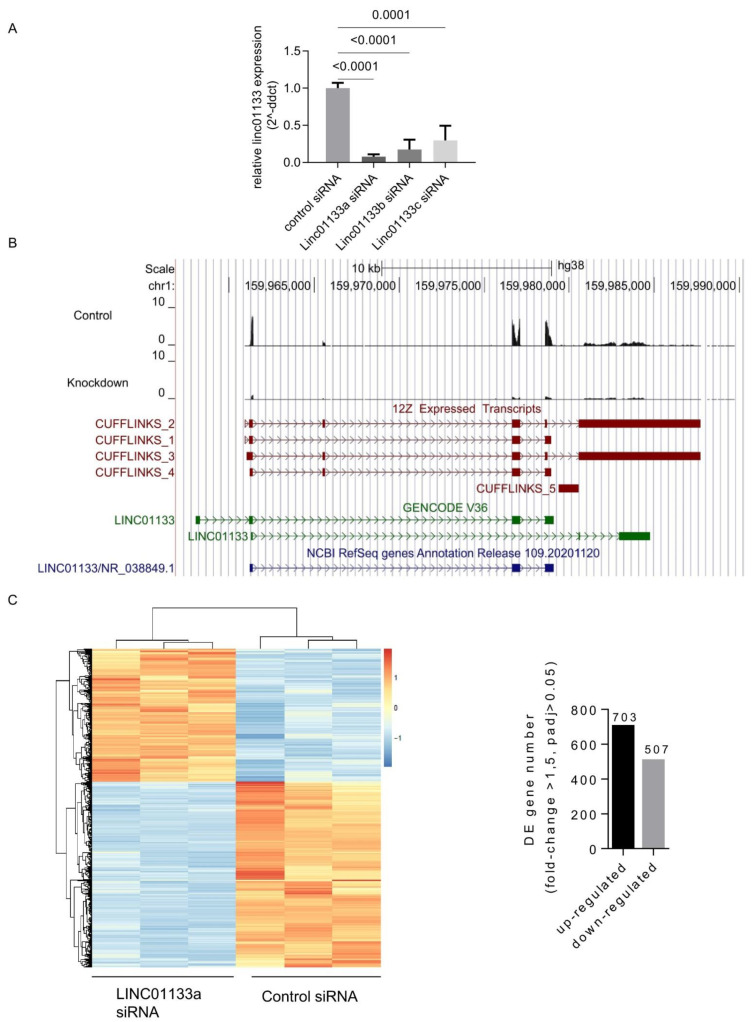
*LINC01133* knockdown leads to the deregulation of hundreds of genes in an endometriosis epithelial cell line. (**A**) qRT-PCR shows relative expression of *LINC01133* is significantly reduced in 12Z cells for all 3 siRNA oligos that were used. (**B**) Tissue-specific *LINC01133* isoforms are efficiently knocked down in 12Z cells. Top: A UCSC genome browser screenshot shows RNA sequencing reads mapping to *LINC01133* are dramatically reduced in a knockdown with siRNA oligo *LINC01133a*. Bottom: Genome assembly using Cufflinks reveals multiple *LINC01133* isoforms in 12Z cells that differ from the annotated GENCODE and Refseq transcripts. (**C**) Left: Hierarchical clustering of differentially expressed genes from RNA sequencing biological replicates shows that the control and *LINC01133a* siRNA samples cluster separately. Right: Hundreds of genes are up- or down-regulated in the *LINC01133a* knockdown in 12Z cells.

To evaluate the role of *LINC01133* within the epithelial cell compartment of endometriosis lesions, we performed transient siRNAs-based knockdown of *LINC01133* in the 12Z endometriosis epithelial cell line, followed by RNA- sequencing. First, using qRT-PCR we confirmed the efficiency of *LINC01133* knockdown using three independent siRNA oligos. Significant knockdown was achieved for all 3 oligos (*p* ≤ 0.0001), with the most efficient siRNA *LINC01133a* reducing *LINC01133* expression by more than 90% 72 h after transfection (Figure 2A). In order to identify any genes and pathways affected by *LINC01133* knockdown, we then conducted RNA-sequencing comparing *LINC01133a* knockdown cells with non-targeting siRNA control cells 72 h after transfection (3 biological replicates each). We identified four *LINC01133* transcript isoforms that are expressed in 12Z cells and confirmed that all were efficiently targeted by the *LINC01133a* siRNA oligo (Figure 2B). Further, analysis revealed 1210 differentially expressed (DE) transcripts in 12Z knockdown cells, compared to controls using a fold change cutoff of >1.5 and adjp < 0.05. From those DE genes, 703 were down-regulated and 507 up-regulated in knockdown cells (Table S4). These transcriptional changes enabled a clear separation of *LINC01133* knockdown and control samples by unsupervised hierarchical clustering (Figure 2C).

### 2.3. The Knockdown of LINC01133 in 12Z Cells Effects Genes and Pathways with a Known Function in Endometriosis Lesion Formation

To gain insight into the function of the genes being regulated by *LINC01133*, we carried out gene ontology enrichment analysis (GOEA) (http://bioinformatics.sdstate.edu/go/) (accessed on 2 December 2020) [26] and gene set enrichment analysis (GSEA) (http://www.gsea-msigdb.org/gsea) (accessed on 2 December 2020) [27,28]. The GOEA showed enrichment in genes that control cell proliferation, migration and angiogenesis (Figure S2A and Table S5), all processes to be involved in the pathogenesis of the disease. GSEA enrichment in targets of the mammalian histone methyltransferase EZH2, adult tissue stem cells and mesenchymal cells (Figure S2B and Table S6), Together these results suggest that *LINC01133* may be involved in the regulation of epithelial cell proliferation, invasion and cell fate conversion (EMT), processes that are known to support ectopic lesion growth.

### 2.4. LINC01133 Regulates Proliferation and Invasion of Endometriosis Epithelial Cells In Vitro

**Figure 3:**
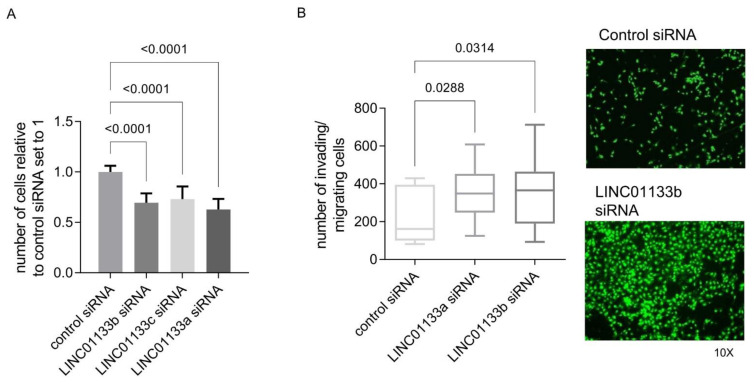
*LINC01133* knockdown reduces proliferation and increases invasion of 12Z endometriosis epithelial cells. (**A**) Relative number of proliferating cells is significantly reduced in *LINC01133* knockdowns using 3 different siRNA oligos. Data are presented as bar plots of mean values from three biological replicates +SD. (**B**) Invading cells are significantly increased following *LINC01133* knockdown using 2 different siRNA oligos. Left: Quantification of the number of invading cells after *LINC01133* knockdown analyzed by the trans-well method. Right: Representative images of control and knockdown cells at 10× magnification stained with CyQuant fluorescence dye (green), which shows fluorescence enrichment when bound to cellular nucleic acids. Data are presented as a box plot ranging from minimum to maximum, including the median and box boundaries at the 25th and 75th percentiles from three biological replicates. Six independent fields were counted and the mean values taken for analysis. Statistical analysis of the data between the groups in A and B was done using Kruskal–Wallis ANOVA test with Dunn’s test for multiple comparison. Adjp < 0.05 were considered significant.

To determine if the phenotypic changes predicted by GOEA and GSEA analysis following *LINC01133* knockdown in 12Z cells occur, we evaluated changes in cell proliferation and invasion in 12Z cells 72 h post knockdown. The results showed that *LINC01133* knockdown leads to a slight, but significant downregulation of 12Z proliferation (Figure 3A) and significantly enhances the invasion phenotype of knockdown cells (Figure 3B). The relative proliferation rate of *LINC01133* knockdown cells was 30% lower (adjp < 0.0001), and the invasion rate was 1.5 times higher (adjp < 0.05) when compared to cells transfected with the siRNA control oligo.

### 2.5. LINC01133 Regulates Cell Cycle and the Levels of Expression of Cell Cycle Regulatory Proteins p21 and Cyclin A

**Figure 4:**
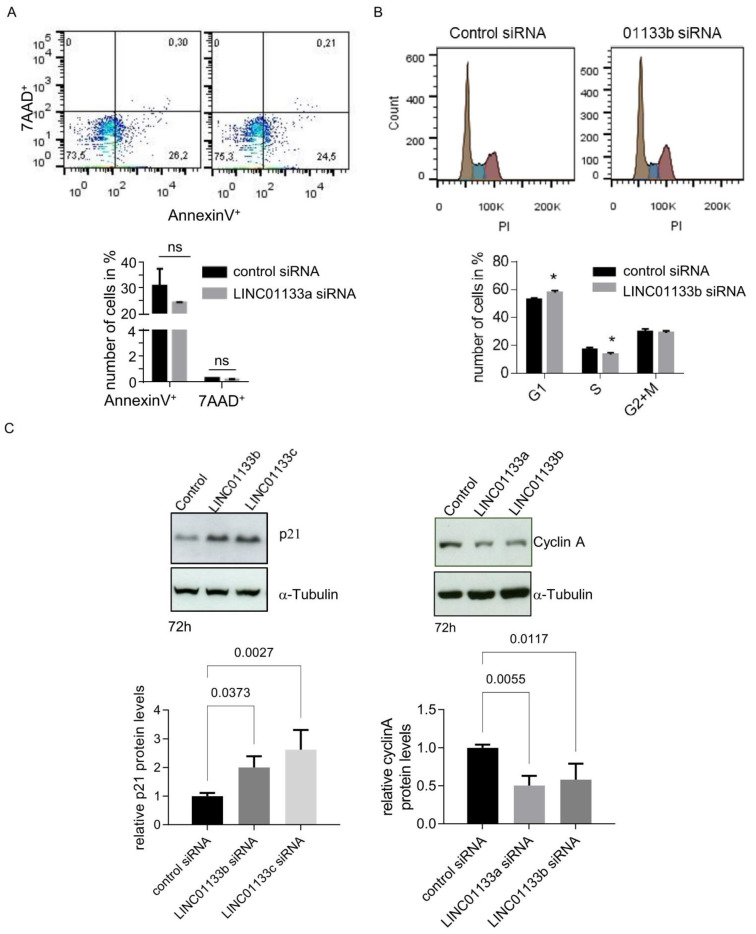
*LINC01133* knockdown leads to an increase in cells in the G1 phase and a decrease in cells in the S phase associated with an increase in p21 and a decrease in Cyclin A levels. (**A**) No significant changes in the number of apoptotic cells are seen 72 h after *LINC01133* knockdown. Top: Representative flow cytometry scatter plots for AnnexinV+ versus 7-AAD+ cells. Bottom: No significant change in the number of AnnexinV+ or 7AAD+ cells was observed between *LINC01133a* knockdown and control siRNA transfected cells. Mean values + SD of three biological replicates are shown. (**B**) *LINC01133* knockdown leads to an increase in cells in the G1 phase and a decrease in cells in the S phase. Top: Representative DNA profiles obtained from flow cytometry analysis of PI stained 12Z cells 72 h after transfection with *LINC01133b* siRNA or control siRNA (brown G1 peak, blue S phase, purple G2 + M). Bottom: The percentage of cells in each cell cycle phase are plotted as mean + SD of three independent experiments. Statistically significant differences between the groups in A and B are indicated with a star on the top of each panel. *—adjp < 0.05 (two-way ANOVA test with Sidak’s for multiple comparison), ns-not significant. (**C**) p21 protein is significantly upregulated and Cyclin A protein significantly down-regulated following *LINC01133* knockdown. Top: Representative examples of Western blot analysis following *LINC01133* knockdown of p21 (left) and Cyclin A (right) together with the α-tubulin loading control. Bottom: Densitometric analysis of p21 (left) and cyclin A (right) levels from Western blots normalized to the α-tubulin loading control. Data are displayed as bar graphs with the level from the control siRNA set to 1, and mean and + SD of biological triplicates shown. Statistically significant differences between the groups are indicated with adj *p*-values on the top of each graph (ANOVA, with Dunnett’s multiple comparison test).

We further examined the viability of 12Z cells following *LINC01133* knockdown using an AnnexinV/Propidium Iodide FACS assay. We found that *LINC01133* knockdown did not influence the survival of the cells (Figure 4A). The mean percent of early apoptotic AnnexinV positive cells was about 25% for both cells transfected with a control or *LINC01133a* oligo. Analysis of the DNA profiles of the *LINC01133* siRNA transfected cells showed a slight but significant enrichment of the number of cells in the G1 phase (7%, adjp < 0.005), and a concomitant down-regulation of the number of cells entering S-phase of the cell cycle (5%, adjp < 0.05), compared to control siRNA transfected cells (Figure 4B). This effect on cell cycle in knockdown cells was associated with significant up-regulation of the levels of expression of the cell cycle checkpoint regulatory protein p21 (~2.5-fold, adjp < 0.005) and down-regulation of Cyclin A (~2-fold, adjp < 0.05) (Figure 4C), compared to control oligo transfected cells. These findings were consistent with the results of our RNA-seq (see Table S4), further indicating that changes in expression of these genes may be responsible for the cell cycle phenotype.

### 2.6. LINC01133 Is Not a Regulator of EMT in 12Z Endometriosis Epithelial Cells

The enrichment of differentially expressed genes associated with a mesenchymal phenotype (Figure S2, Table S6A,B) and an increased invasion of 12Z cells following *LINC01133* knockdown (Figure 3B) indicated that these cells may be converted to a more mesenchymal phenotype. To evaluate the role of *LINC01133* in epithelial to mesenchymal transition (EMT) in endometriosis, we further investigated expression of selected EMT regulatory proteins indicated to be differentially expressed in our RNA-seq data (Table S7). qRT-PCR and Western blot analysis confirmed the loss of CDH1 (E-cadherin) following *LINC01133* knockdown (Figure S3A). The levels of *CDH1* transcription correlated with the efficiency of the *LINC01133* knockdown in 12Z cells (Figure 2A, Figure S3A left panel), further supporting the involvement of *LINC01133* in its regulation. Specifically, a knockdown of *LINC01133* to about 10% of the levels of controls (*LINC01133a* siRNA, adjp < 0.0001), led to reduction of the relative levels of *CDH1* expression to 20% of the controls (adjp < 0.0001), whereas *LINC01133* knockdown to 35% (*LINC01133b* siRNA, adjp < 0.0001) led to a 30% reduction of *CDH1* transcript (*p* = 0.007) compared to controls. However, we did not see a classical EMT-associated Cadherin switch in *LINC01133* knockdown cells. The levels of *CDH2* (*N-cadherin*) transcript were downregulated to 53% of normal level in cells with high efficiency of *LINC01133* knockdown (*LINC01133a* oligo, adjp = 0.002), but were not changed in cells with a less efficient knockdown of *LINC01133* (*LINC01133b* siRNA oligo, adjp > 0.05) (Figure S3A, left panel). Further, we validated the significant downregulation of *VCAM-1* (*p* = 0.029, Figure S3B), and significant upregulation of *SOX4* (*p* = 0.02, Figure S3C) and *TGFβ2* (*p* = 0.0014, Figure S3D) in *LINC01133* knockdown cells compared to controls for both the *LINC01133a* and *LINC01133b* oligos. However, we could not validate the reduction of *KRT7* and *KRT19* (Figure S3E,F) in cells with *LINC01133* knockdown, when compared to siRNA controls. Given that we did not see a classic E-cadherin to N-cadherin switch, and that expression changes for some EMT markers could not be validated, these data suggest that *LINC01133* does not play a significant role in regulating EMT in endometriosis epithelial cells.

### 2.7. Active Cytoskeleton Remodeling in 12Z Cells Following LINC01133 Knockdown

**Figure 5:**
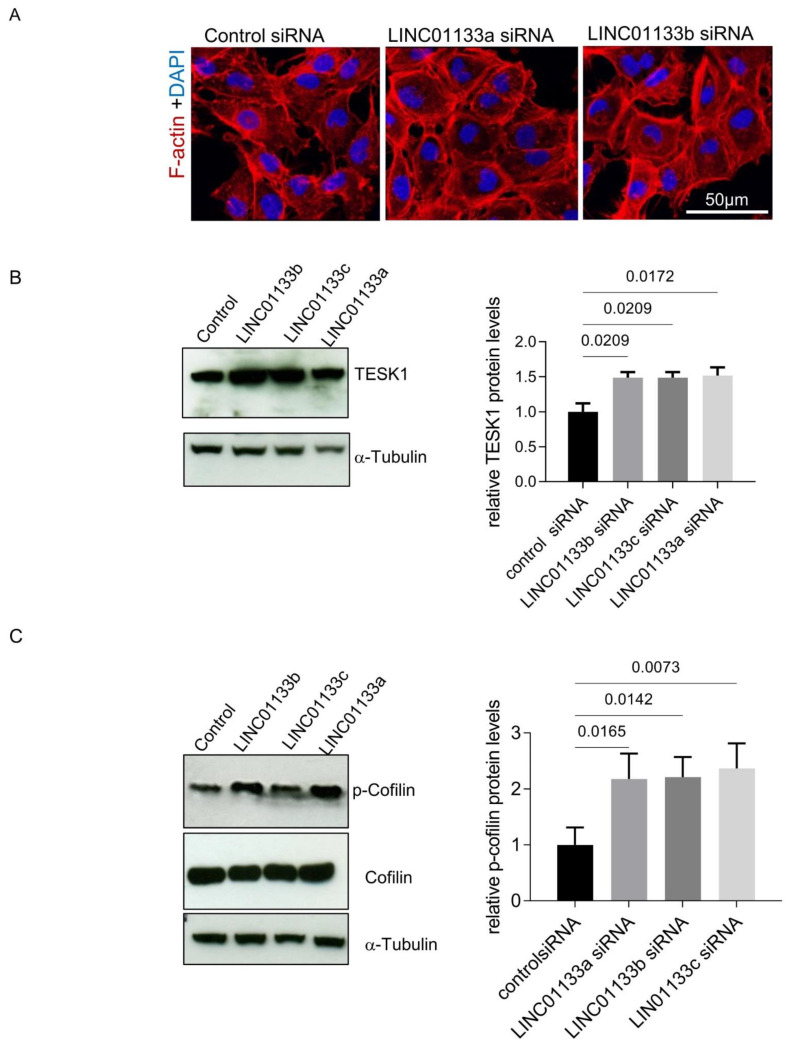
*LINC01133* knockdown affects cellular morphology of 12Z cells (**A**) Immunofluorescence of F-actin 72 h after transfection with siRNA control and 2 different *LINC01133* oligos shows a more flattened phenotype and an increase in the number of actin stress fibers. Representative confocal images are shown (three independent experiments were analyzed). Cell nuclei were visualized with DAPI. The magnification scale of 50 µm is indicated with white line on the figure. (**B**) *LINC01133* knockdown leads to upregulation of the levels of TESK1 protein. Left: Representative Western blot showing TESK1 levels together with the α-tubulin loading control. Right: TESK1 relative protein levels normalized to α-tubulin determined by densitometric analysis of Western blots. The relative levels of TESK1 from three independent experiments are shown as bar graphs with the control set to 1 and the mean and + SD of protein shown (**C**) *LINC01133* knockdown increases the levels of Cofilin phosphorylation. Left: Representative example of *p*-Cofilin and Cofilin levels detected by Western blot with α-tubulin loading control. Right: Phosphorylated Cofilin relative levels normalized to α-tubulin determined by densitometric analysis of Western blots. The relative levels of phosphorylated Cofilin from three independent experiments are shown as bar graphs with the control set to 1, and the mean and +SD of protein shown. Statistically significant differences between the groups are indicated by adj *p*-values on the top of each graph (ANOVA, with Dunnett’s multiple comparison test).

*LINC01133* knockdown led to the deregulation of genes involved in cell adhesion and EMT, and was associated with changes in the cellular phenotype of 12Z knockdown cells, which appeared to have a more flattened, larger phenotype with an increased number of actin stress fibers (Figure 5A). This was supported by analysis of cell area and fluorescence intensity in *LINC01133* knockdown cells compared to controls. We confirmed that the cross-sectional cellular area of *LINC01133* knockdown cells was greater, with knockdown cells 1.7 fold the size of control siRNA treated cells (adjp = 0.006, Figure S4A). Further, analysis of Phalloidin fluorescence intensity showed that *LINC01133* knockdown cells had 4.5-fold higher corrected total cell fluorescence (CTCF) than cells transfected with control siRNA oligo (*p* < 0.0001) (Figure S4B). This data indicated that active actin remodeling may occur following *LINC01133* knockdown. Therefore, we further evaluated the expression and/or activity of some proteins involved in the regulation of actin filaments, stress fibers formation and focal adhesions such as TESK1 and Cofilin. TESK1 phosphorylates and thereby inactivates the Actin severing protein Cofilin at Ser3, thus regulating the organization of the Actin cytoskeleton [29]. We identified *TESK1* as being differentially expressed in 12Z knockdown cells by RNA-seq (Table S4) and confirmed that the protein was significantly increased following *LINC01133* knockdown (1.5-times higher, adjp < 0.05) (Figure 5B). These changes in TESK1 expression were associated with a significant increase in Cofilin phosphorylation to 2.2-fold higher (adjp < 0.05) following *LINC01133* knockdown (Figure 5C). Together this data indicates that *LINC01133* may regulate actin remodeling in endometriosis epithelial cells via this pathway.

## 3. Discussion

LncRNAs are epigenetic regulators that have been implicated in development and disease, but whose role in the pathogenesis of endometriosis remains relatively unknown. Endometriosis shares some features with cancer, including EMT, therefore we chose to investigate the role of *LINC01133* in endometriosis, a well-characterized lncRNA that has been associated with EMT in cervical [21], breast [22], colorectal [23] and gastric [24] cancer. We found that *LINC01133* is significantly upregulated in ectopic endometriosis lesions, and that knockdown in an epithelial endometriosis cell line indicates that it promotes cell proliferation and suppresses cell migration and invasion in endometriosis, but that it does not regulate EMT in this disease. Our results indicate that *LINC01133* affects cell proliferation by affecting the cell cycle via the p21/cyclin pathway, and cellular invasion and cytoskeleton remodeling due to Cofilin phosphorylation and inactivation by the TESK1 kinase. A caveat of our study is that we used the immortalized endometriosis epithelial cell line 12Z for our functional experiments, although this cell line is widely accepted in the field as a cell model of endometriosis [30].

The effects on cell proliferation were associated with cell cycle arrest in G1 and impaired S-phase entry due to significant up-regulation of cell cycle checkpoint protein p21 and concomitant downregulation of Cyclin A. The mechanism by which *LINC01133* may regulate these genes in endometriosis remains unclear. In non-small cell lung carcinoma *LINC01133* suppresses the transcription of *CDKN1A* (*p21*) via a direct EZH2-mediated chromatin remodeling mechanism [31]. In another context, *LINC01133* promotes the progression of cervical cancer by sponging miR-4784 to cause the up-regulation of AT-hook DNA-binding motif-containing protein 1 (AHDC1) promoting EMT [21]. The high basal level of p21 expression and moderate transcriptional activation upon *LINC01133* knockdown (~2.5-fold) indicates that sponging rather than an EZH2 mediated p21 activation may be a more likely mechanism of regulation, although this remains to be tested.

Impaired expression of *LINC01133* has been associated with the regulation of EMT in cancer [23,31,32]. EMT is a multi-stage process leading to the gradual remodeling of the epithelial into a mesenchymal phenotype. This includes the loss of epithelial markers and concomitant acquisition of mesenchymal markers, an increase in cell migration and invasion, disruption of cell-cell contacts, impaired adhesion and the remodeling of the cytoskeleton [19]. This molecular remodeling also takes place during the establishment of endometriosis lesions [17]. However, although we saw an enrichment of some mesenchymal gene sets among differentially expressed genes in 12Z endometriosis epithelial cells following *LINC01133* knockdown, we found little phenotypic evidence for EMT. Our data showed a significant upregulation of *TGFβ2* following *LINC01133* knockdown is associated with an increase in the levels of expression of the master regulator of EMT, *SOX4* [33,34] and subsequent down-regulation of *CDH1* and *CDH2*. However, expression of the epithelial markers *KDR7* and *KDR19* [35], along with the EMT regulators *TWIST*, *SNAIL*, *ZEB1* and *ZEB2* [36] were not significantly affected by *LINC01133* knockdown. CDH1 is a tumor suppressor and cell polarity regulator [37] and the loss of CDH1 promotes motility and invasion. There is also some evidence that in endometriosis lesions the loss of CDH2 expression may be associated with increased invasive capacity of endometrial epithelial cells. Matsuzaki et al. [38] have shown that deep infiltrating endometriosis lesions express less CDH2, compared to early peritoneal lesions. In normal endometrial tissue high levels of CDH2 were associated with the proliferative phase of the cycle [39]. However, whether activation of CDH2 by *LINC01133* is responsible for the loss of proliferation capacity and increased invasiveness of endometriosis epithelial cells needs to be tested.

Recently, we have shown that the levels of expression of *VCAM-1* are increased in tissue samples of women with endometriosis, compared to women without the disease [40]. The loss of *VCAM-1* was shown to attenuate the TGF-β1 induced proliferation, migration and invasion of endometriosis stroma cells derived from ovarian endometriomas [41]. Our data showed that the function of the protein as a regulator of cell invasion of epithelial endometriosis cells might differ from those in stroma, while downregulation of *VCAM-1* following *LINC01133* knockdown was associated with an increase of cellular invasion. As up-regulation of *VCAM-1* in malignant cells is associated with recruitment of tumor-associated monocytes and macrophages and immune escape of the tumors [42,43], we postulate that *LINC01133* dependent *VCAM-1* regulation in endometriosis epithelial cells may be related to immune surveillance of the lesions.

A central event in cellular invasion is the dynamic cytoskeleton remodeling leading to changes in cellular morphology. We [44] and others [45,46] have shown that the dysregulation of cytoskeleton dynamics and related signaling pathways are linked to pathogenesis of endometriosis and disease-associated infertility. Actin filaments, microtubules, and intermediate filaments involved in the formation of cytoskeletal structures, such as stress fibers and pseudopodia promote the invasion of normal cells and invasion and metastasis of tumor cells. Here we showed that the increase in the invasion capacity of 12Z cell under *LINC01133* knockdown is associated with changes in cellular morphology to more flattened and larger phenotype with an increased number of stress fibers, provoked by the activation of TESK1 expression and inactivation of Cofilin. TESK1 is a serine/threonine kinase with kinase domain similar to those of LIM-kinases and a unique C-terminal proline-rich domain [47] known to phosphorylate Cofilin at Ser-3 [29]. Cofilin plays an essential role in actin filament dynamics by enhancing depolymerization and severance of actin filaments [48]. These activities of Cofilin are abolished by phosphorylation at Ser-3. Therefore, the changes in Cofilin phosphorylation at Ser-3 are regarded as one of the most important mechanisms for regulating Cofilin activity and actin filament dynamics. It has been shown that induction of stress fibers require an active Rho-ROCK signaling pathway independent of TESK1 [29]. Several studies also indicate that the balance between Rho and Rac activity in cells determines the patterns of actin organization, cell morphology and motility [49]. Thus, the effects of *LINC01133* on the cellular filament and cytoskeleton dynamics may not be restricted only to the TESK1/Cofilin pathway.

Overall, in this study, we found that *LINC01133* is overexpressed in ectopic endometriosis lesions compared to the eutopic endometrium of women both with and without the disease. By knocking down *LINC01133* in endometriosis epithelial cells we were able to show that the lncRNA promotes cellular proliferation and inhibits cell invasion in these cells, and to identify components of these pathways that were affected, including p21, Cyclin A and TESK. These results indicate that *LINC01133* may be a clinically relevant player in endometriosis, although this remains to be tested in vivo.

## 4. Materials and Methods

### 4.1. Study Population

For this study, tissue samples were collected in accordance with the protocols of the Endometriosis Marker Austria (EMMA) study, a prospective cohort study conducted at the Tertiary Endometriosis Referral Center of the Medical University of Vienna. Premenopausal women 18–50 years of age undergoing a laparoscopic procedure due to suspected endometriosis, infertility, chronic pelvic pain, benign adnexal masses or uterine leiomyoma were invited to participate in the EMMA study. Women who had acute inflammation, known or suspected infectious disease, chronic autoimmune disease or malignancy were excluded from the study. Ethics approval for this study was provided by the institutional ethics committee of the Medical University of Vienna (EK 545/2010). Verbal and written informed consent was obtained from each participant prior to inclusion into the study. The detailed baseline characteristics of the participants are summarized in Table S1. Briefly, from a total number of *n* = 95 participating women, *n* = 42 were defined as controls and *n* = 53 were patients suffering from endometriosis. The control group consisted of women undergoing laparoscopy for uterine fibroids, benign ovarian cysts, fallopian tube disorders or diagnostic laparoscopy due to unexplained infertility or chronic pelvic pain. Each participating woman contributed only one sample of eutopic endometrium and some of the women with endometriosis contributed samples of diverse types of endometriotic lesions. All tissue samples were collected during laparoscopic surgical intervention for diagnosis and/or therapy of endometriosis. All samples were collected in accordance to Endometriosis Phenome and Biobanking Harmonization Project guidelines [50].

### 4.2. Cell Line for In Vitro Evaluation of LINC01133 Function

Endometriotic epithelial cell line 12Z established and characterized by the laboratory of Professor Starzinski-Powitz [51,52] was kindly provided for our in vitro studies. The cells were cultured in Dulbecco’s modified Eagle’s medium (DMEM-F12) containing penicillin/streptomycin in final concentrations of 50 U/mL and 50 µg/mL, respectively and 10% *v*/*v* fetal calf serum (FCS). The cells were maintained in a 37 °C CO_2_-humified incubator. Cells were tested and found to be negative for mycoplasm infection. All cell culture reagents were purchased from Thermo Fisher Scientific (Waltham, MA, USA) or Sarstedt (Nümbrecht, Germany).

### 4.3. RNA-Scope

To visualize the subcellular localization of *LINC01133* RNA in tissue samples of women with endometriosis we used the RNAscope^®^ 2.5 HD Red assay on formalin-fixed paraffin-embedded eutopic endometrial tissues of women with endometriosis (*n* = 5), according to the manufacturer’s protocol (Advanced Cell Diagnostics (ACD), Hayward, CA, USA). This method uses a signal amplification method and double Z probe design provides high sensitivity and specificity suitable for detecting relatively lowly expressed RNAs, such as lncRNAs. The system visualizes target RNA as a single dot, where each dot is an amplified signal of an individual RNA molecule. We used the probe Hs-LINC01133 designed by ACD to specifically detect *LINC01133*, and probe dapB (bacterial dihydrodipicolinate reductase, PN310043) as a negative control.

### 4.4. LINC01133 Knockdown

The 12Z cells were seeded in complete culture cell medium 12 h prior to transfection on 6-well culture plates (Nunc, Thermo Fisher Scientific, Waltham, MA, USA) at a concentration of 1 × 10^5^ cells/well and allowed to grow to approximately 40% confluency. The cells were then transfected with one of three different LINC0133 targeting siRNA oligos at a final concentration of 10 nM (Table S2A) or a non-targeting control siRNA oligo (Cat.4390846, Ambion, Austin, Texas, USA) using Lipofectamine RNAiMAX transfection reagent according to the manufacturer’s protocol (Invitrogen by Life Technology, Waltham, MA, USA). Phenotypic analysis of *LINC01133* knockdown cells for changes in cellular proliferation and invasion/migration was conducted 48 h post-transfection. RNA-sequencing, qRT-PCR and Western blot analysis were conducted 72 h post-transfection. The 3 siRNAs had similar *LINC01133* knockdown efficiencies (Figure 2A). Therefore, for each experiment, we first checked *LINC01133* knockdown efficiency by qRT-PCR, and then proceeded with the 2 siRNAs that showed the greatest knockdown efficiency.

### 4.5. RNA Isolation

Frozen tissue samples were homogenized with a Precellys 24 homogenizer (PEQLAB, Erlangen, Germany). Subsequently, total RNA was isolated from eutopic and ectopic endometrium using the Agilent Absolutely RNA kit in accordance with the manufacturer’s instructions (DNase I treatment included), and total RNA from the 12Z cell line was isolated using the RNeasy mini kit (Qiagen). To remove DNA contamination the RNA samples were subsequently treated with DNAseI using RapidOut DNA removal Kit (Thermo Fisher Scientific, Waltham, MA, USA). RNA concentration and purity were determined by measuring optical density using a NanoDrop ND-1000 spectrophotometer (NanoDrop Technologies, Wilmington, DE, USA). We defined the quality of the RNA samples to be sufficient when the ratios of OD260/280 and OD260/230 were around 2.00. Additionally, for RNA-sequencing we confirmed that RNA integrity was RIN > 7 on a Bio Analyzer (Agilent Technologies, Santa Clara, CA, USA).

### 4.6. RNA-Sequencing and Data Analysis

RNA sequencing was performed by the Next Generation Sequencing Facility at Vienna BioCenter Core Facilities (VBCF), a member of the Vienna BioCenter (VBC), Austria. From the total RNA, we provided they conducted poly-A enrichment for mRNAs and prepared libraries using the Illumina TruSeq RNA kit. The six libraries (3 control oligo, 3 *LINC01133* knock-down) were multiplexed on a single lane and subjected to 125 bp paired-end sequencing on an Illumina HiSeq2500 machine. The facility provided de-multiplexed BAM files containing the raw reads, which we converted to fastq using Samtools (v1.10) for alignment with STAR using the parameters: --outFilterMultimapNmax 1 --outSAMstrandField intronMotif --outFilterIntronMotifs RemoveNoncanonical --outSAMtype BAM SortedByCoordinate --quantMode GeneCounts. On average 38M reads (94% of all reads) mapped uniquely to the human genome (Table S2B). Annotation files and genome sequences were downloaded from https://www.gencodegenes.org (accessed on 2 December 2020). Index for alignments was prepared using STAR (2.7.6a_patch_2020-11-17) [53] with FASTA files from the GRCh38.p13 assembly and Gencode (v36) gene annotation in GTF format.

Differential gene expression analysis was performed in the R statistical computing environment (v4.0.3) [54] using the DESeq2 package (v1.30.0) [55] with count tables produced by STAR during alignment (*ReadsPerGene.out.tab files).

### 4.7. Assembly of LINC01133 Isoforms Expressed in the 12Z Cell Line

Aligned reads from the genomic positions chr1:159955239-15998963, were extracted using Samtools for all downstream analyses [56]. For that region, read coverage was calculated using bam2wig (v4.0.0) (https://github.com/MikeAxtell/bam2wig) accessed on 3 December 2020. Read coverage at each informative genomic position was normalized for sequencing depth and averaged over all control siRNA transfected and knockdown *LINC01133a* siRNA samples. Transcript assembly was performed using Cufflinks (v2.2.1) [57] with the parameter: -F 0.05. Note that for transcript assembly only the control siRNA samples were used and the resulting GTF files were merged using Cuffmerge to obtain the final *LINC01133* annotation.

### 4.8. Gene Set Enrichment Analysis (GSEA) and Gene Ontology Enrichment Analysis (GOEA)

To test whether the differentially expressed genes identified by our cutoff criteria (fold change > 1.5, adjusted *p*-value (adjp) < 0.05) are associated with specific biological functions we performed gene ontology enrichment analysis (GOEA http://bioinformatics.sdstate.edu/go/) (accessed on 25 November 2020) [26] and gene set enrichment analysis (GSEA, Broad Institute http://www.gsea-msigdb.org/gsea) [27] accessed on 26 December 2020) [28]. For GOEA annotation Fisher’s exact test is used to determine if different annotation terms are enriched among the differentially expressed genes. Gene ontology (GO) terms showing a Fisher’s exact *p*-value < 0.05 were considered significantly enriched. To calculate GSEA we used the Molecular Signature Database (MSigDB) to investigate the overlap between our gene lists and known annotated gene sets. Gene sets showing a false discovery rate (FDR) <0.05 were considered as significantly enriched among differentially expressed genes. We considered the biological processes associated with significantly enriched GO terms or MSigDB gene sets as being potentially relevant for endometriosis.

### 4.9. Quantitative Reverse Transcription PCR (qRT-PCR) for Measuring mRNA Expression

Total RNA was reverse transcribed with SuperScript^®^ III First-Strand Synthesis Reverse Transcriptase using a mixture of oligo-d (T) and random hexamer primers (Life Sciences Advance Technology, St. Petersburg, FL, USA). These cDNA preparations were then diluted 2 fold with water before being assayed. qRT-PCR was performed in triplicate in 96-well optical plates with 6 biological replicates. Each reaction contained 1X TaqMan PCR master mix (Applied Biosystems, Waltham, MA, USA with ROX reference dye) and 0.2 μM of each specific primer pair-probe set listed in Table S3. qRT-PCR was performed using a 7500 Fast Real-Time PCR System (Applied Biosystems, Waltham, MA, USA), with an initial denaturation for 10 minutes at 95 °C, primer annealing at 50 °C for 2 min, followed by 40 cycles of 15 seconds at 95 °C and 1 minute at 60 °C. The relative expression of target genes was calculated using the delta-CT method as described [58], and normalized to *GAPDH* expression. The average Ct values were ≤ 30 except for *SOX4* transcripts that showed an average Ct-value of 35 cycles.

### 4.10. Protein Isolation and Western Blot

Cells for Western Blot analysis were harvested and lysed in a whole-cell lysis buffer composed of 1% Triton-X 100, 10 mM Tris-HCl, pH7.4, 150 mM NaCl and 5 mM EDTA. Prior to use, the lysis buffer was supplemented with phosphatase and protease inhibitor cocktail (phosStop, cOmplete mini, EDTA free, Thermo Fisher Scientific, Waltham, MA, USA). The protein concentration was determined by the standard Bradford assay. The normalized samples were immunoblotted as previously described [59] and incubated with primary antibodies for the proteins of interest (Table S3). The secondary antibodies were diluted in a Tris pH 8.0, 0.1% Tween 20 buffer and incubated for 1 h at room temperature. Bound antibodies were detected by the horseradish peroxidase chemiluminescent substrate Luminata^TM^ (Millipore Corporation, Burlington, MA, USA). X-ray films (GE Healthcare, Frankfurt am Main, Germany) were used for chemiluminescence detection. The levels of protein expression on the blot were quantified using ImageJ Software (http://rsbweb.nih.gov/ij) (accessed on 15 March 2021).

### 4.11. Analysis of Cell Cycle and Cell Death Using Fluorescence-Activated Cell Scanning (FACS) Flow Cytometry

For FACS based analysis of the cell cycle changes upon *LINC01133* knockdown, we used standard propidium iodide (PI) DNA staining protocol. In brief, transfected cells were harvested 72 h after siRNA knockdown and 1 × 10^6^ cells were fixed in 70% precooled ethanol for 2 h on ice. After washing with PBS (Thermo Fisher Scientific, Waltham, MA, USA) the cells were re-suspended in 0.5 mL PI/RNAse containing staining buffer (550825), BD Pharminogen™, Heidelberg, Germany) supplemented with 10 μL PI staining solution (51-6621-1E, BD Pharmingen™, Heidelberg, Germany). After incubation for 15 minutes at room temperature, the number of PI positive cells was measured by flow cytometry. The effect of *LINC01133* on cellular apoptotic rate was evaluated using staining with AnnexinV (FITC-conjugated antibody (640906), BioLegend, San Diego, CA, USA; Annexin V Binding Buffer, (422201), BioLegend, San Diego, CA, USA) in conjunction with the vital dye 7-amino-actomycin D (00-6993-50) eBioscience, San Diego, CA, USA) followed by flow cytometry measurement. A total of 1 × 10^6^ cells 72 h after transfection was used in the analysis, with a total of 10,000 events recorded for each sample, and unstained cells being used as assay control.

### 4.12. Proliferation Assay

The proliferation rate of 12Z cells 72 h after LINC01133 knockdown was analyzed using the CyQuant direct cell proliferation Assay (Invitrogen, Waltham, MA, USA) according to the manufacture’s protocol. Prior to the assay, transfected cells were trypsinized 48 h after transfection with targeting and control siRNA oligos, seeded on 96 flat-bottom cell culture plates (Thermo Fisher Scientific, Roskilde, Denmark) at a concentration of 15,000 cells/well and allowed to grow for an additional 12 h. The level of fluorescence was accessed with the Clariostar^plus^ microplate reader (BMG Labtech, Ortenberg, Germany) with filters appropriate for 480 nm excitation and 520 nm emission maxima. The observed fluorescence values were first corrected for the background fluorescence determined with a cell- free sample, and a standard curve was used to estimate cell number. All measurements were performed in technical triplicates, and the average number of proliferating cells relative to control siRNA-treated cells was set to 1.

### 4.13. Matrigel-Invasion Assay

The ability of cells to migrate or invade through a Matrigel barrier was measured in a Boyden chamber assay with polycarbonate membranes. After 48 h of siRNA knockdown, an equal number of 12Z cells (2 × 10^4^) were re-suspended in 100 µL of growing media supplemented with 1% *v*/*v* FCS and antibiotics and plated on top of matrigel coated filter (Corning Matrigel growth factor reduced (354230); Corning Incorporated, Corning, NY, USA, 1% matrigel solution in PBS, filter: 6.5 mm diameter, 8 µm-pores, Corning Incorporated, Corning, NY, USA). The cells were allowed to migrate/invade for 12 h toward the bottom of the well, which contained media supplemented with 10% FCS. Cells on the lower surface of the filter were fixed with 4% PFA and stained with CyQUANT™ Direct Red nucleic acid stain (Invitrogen, Thermo Fisher Scientific, Waltham, MA, USA) and photographed with an ×10 objective under the microscope. The values for invasion were taken as the average number of invaded cells per photographic field over five independent fields per experiment and expressed as averages of triplicate experiments.

### 4.14. Immunofluorescence, Cell Size and Stress Fiber Analysis

12Z cells were seeded in 6-well plates and subject to siRNA knockdown for a non-targeting control, *LINC01133a* and *LINC01133b* oligos, as described above. The cells were trypsinized 48 h after transfection, 20,000 cells from each treatment re-plated on 8-well chamber slides, and then 24 h later the cells were fixed and processed for immunofluorescence as previously described [60]. Cells were stained for F-actin (Rhodamine conjugated Phalloidin, Invitrogen Cat. R415) and counterstained for DAPI. Cells were then imaged with a Leica SP8 confocal microscope using an ×63 glycerol objective and images processed using ImageJ.

We measured cell area in ImageJ using a previously described protocol (https://www.youtube.com/watch?v=IeicxaeMUwA) (accessed on 15 March 2021). First, we used the line tool to draw a line over a scale bar on one image and selected “measure” under the analyze menu. Next, we selected “set scale” under the analyze menu, and entered the number of pixels for 20 µM, a known distance of 20, and µM as the unit of length, and ticked the “Global” box so that this scale would be applied to all images. To measure the cross-sectional area of cells we chose the free-hand selection tool, mapped the outline of the first cells with the computer mouse, and pressed “measure” under the analyze menu. We then repeated this for all cells in each image.

We used ImageJ to measure fluorescence intensity in control and *LINC01133a* knockdown cells followed an established technique https://theolb.readthedocs.io/en/latest/imaging/measuring-cell-fluorescence-using-imagej.html (accessed on 15 March 2021) [61]. Briefly, we used drawing tools to select cells, chose “area integrated intensity” and “mean grey value” in the “set measurements” menu, and then “measure” from the analyze menu. We then measured an area near the cell with no fluorescence as a background control. We then repeated this process until all cells in the field had been measured. We then calculated the corrected total cell fluorescence (CTCF) for each cell in each image using the following formula (Equation (1)):

CTCF = Integrated Density − (area of selected cell × mean fluorescence background) (1)

### 4.15. Statistics

All statistical tests were performed using SPSS version 27.0 (IBM, SPSS statistics 27.0, Armonk, NY, USA: IBM Corp.) for patient cohort characterization and Prism (GraphPad Prism 9.0 software, La Jolla, CA, USA) for the remaining experimental settings. The exact statistical procedures for each analysis are described in the corresponding figure legends.

### 4.16. Websites and Software

The following websites were used for analysis and to download software for this study: http://bioinformatics.sdstate.edu/go/, http://www.gsea-msigdb.org/gsea, https://www.gencodegenes.org, https://github.com/MikeAxtell/bam2wig, https://www. R-project.org/ (accessed on 25 November 2020).

## Data Availability

RNA-sequencing data and processed files from this study are available from GEO database under the accession number GSE174741 (https://www.ncbi.nlm.nih.gov/geo/query/acc.cgi?acc=GSE174741) (accessed on 15 March 2021).

## Supplementary Materials

The following are available online at https://www.mdpi.com/article/10.3390/ijms22168385/s1.

## References

[1] Zondervan K.T., Becker C.M., Missmer S.A. (2020). Endometriosis. N. Engl. J. Med..

[2] Hogg C., Horne A.W., Greaves E. (2020). Endometriosis-Associated Macrophages: Origin, Phenotype, and Function. Front. Endocrinol..

[3] Kapranov P., Cheng J., Dike S., Nix D.A., Duttagupta R., Willingham A.T., Stadler P.F., Hertel J., Hackermuller J., Hofacker I.L. (2007). RNA maps reveal new RNA classes and a possible function for pervasive transcription. Science.

[4] Frankish A., Diekhans M., Ferreira A.M., Johnson R., Jungreis I., Loveland J., Mudge J.M., Sisu C., Wright J., Armstrong J. (2019). GENCODE reference annotation for the human and mouse genomes. Nucleic Acids Res..

[5] Ponting C.P., Oliver P.L., Reik W. (2009). Evolution and functions of long noncoding RNAs. Cell.

[6] Wang K.C., Chang H.Y. (2011). Molecular mechanisms of long noncoding RNAs. Mol. Cell.

[7] Uszczynska-Ratajczak B., Lagarde J., Frankish A., Guigo R., Johnson R. (2018). Towards a complete map of the human long non-coding RNA transcriptome. Nat. Rev. Genet..

[8] Statello L., Guo C.J., Chen L.L., Huarte M. (2021). Gene regulation by long non-coding RNAs and its biological functions. Nat. Rev. Mol. Cell Biol..

[9] Geisler S., Coller J. (2013). RNA in unexpected places: Long non-coding RNA functions in diverse cellular contexts. Nat. Rev. Mol. Cell Biol..

[10] Sun P.R., Jia S.Z., Lin H., Leng J.H., Lang J.H. (2014). Genome-wide profiling of long noncoding ribonucleic acid expression patterns in ovarian endometriosis by microarray. Fertil. Steril..

[11] Cai H., Zhu X., Li Z., Zhu Y., Lang J. (2019). lncRNA/mRNA profiling of endometriosis rat uterine tissues during the implantation window. Int. J. Mol. Med..

[12] Li Y., Liu Y.D., Chen S.L., Chen X., Ye D.S., Zhou X.Y., Zhe J., Zhang J. (2019). Down-regulation of long non-coding RNA MALAT1 inhibits granulosa cell proliferation in endometriosis by up-regulating P21 via activation of the ERK/MAPK pathway. Mol. Hum. Reprod..

[13] Liu Y., Ma J., Cui D., Fei X., Lv Y., Lin J. (2020). LncRNA MEG3-210 regulates endometrial stromal cells migration, invasion and apoptosis through p38 MAPK and PKA/SERCA2 signalling via interaction with Galectin-1 in endometriosis. Mol. Cell. Endocrinol..

[14] Zhang C., Wu W., Zhu H., Yu X., Zhang Y., Ye X., Cheng H., Ma R., Cui H., Luo J. (2019). Knockdown of long noncoding RNA CCDC144NL-AS1 attenuates migration and invasion phenotypes in endometrial stromal cells from endometriosisdagger. Biol. Reprod..

[15] Qiu J.J., Lin X.J., Zheng T.T., Tang X.Y., Zhang Y., Hua K.Q. (2019). The Exosomal Long Noncoding RNA aHIF is Upregulated in Serum From Patients With Endometriosis and Promotes Angiogenesis in Endometriosis. Reprod. Sci..

[16] Wang X., Zhang J., Liu X., Wei B., Zhan L. (2021). Long noncoding RNAs in endometriosis: Biological functions, expressions, and mechanisms. J. Cell. Physiol..

[17] Proestling K., Birner P., Gamperl S., Nirtl N., Marton E., Yerlikaya G., Wenzl R., Streubel B., Husslein H. (2015). Enhanced epithelial to mesenchymal transition (EMT) and upregulated MYC in ectopic lesions contribute independently to endometriosis. Reprod. Biol. Endocrinol..

[18] Zondervan K.T., Becker C.M., Koga K., Missmer S.A., Taylor R.N., Vigano P. (2018). Endometriosis. Nat. Rev. Dis. Primers.

[19] Konrad L., Dietze R., Riaz M.A., Scheiner-Bobis G., Behnke J., Horne F., Hoerscher A., Reising C., Meinhold-Heerlein I. (2020). Epithelial-Mesenchymal Transition in Endometriosis-When Does It Happen?. J. Clin. Med..

[20] Yang W., Yue Y., Yin F., Qi Z., Guo R., Xu Y. (2021). LINC01133 and LINC01243 are positively correlated with endometrial carcinoma pathogenesis. Arch. Gynecol. Obstet..

[21] Feng Y., Qu L., Wang X., Liu C. (2019). LINC01133 promotes the progression of cervical cancer by sponging miR-4784 to up-regulate AHDC1. Cancer Biol. Ther..

[22] Song Z., Zhang X., Lin Y., Wei Y., Liang S., Dong C. (2019). LINC01133 inhibits breast cancer invasion and metastasis by negatively regulating SOX4 expression through EZH2. J. Cell Mol. Med..

[23] Kong J., Sun W., Li C., Wan L., Wang S., Wu Y., Xu E., Zhang H., Lai M. (2016). Long non-coding RNA LINC01133 inhibits epithelial-mesenchymal transition and metastasis in colorectal cancer by interacting with SRSF6. Cancer Lett..

[24] Yang X.Z., Cheng T.T., He Q.J., Lei Z.Y., Chi J., Tang Z., Liao Q.X., Zhang H., Zeng L.S., Cui S.Z. (2018). LINC01133 as ceRNA inhibits gastric cancer progression by sponging miR-106a-3p to regulate APC expression and the Wnt/beta-catenin pathway. Mol. Cancer.

[25] Yang Y.-M., Yang W.-X. (2017). Epithelial-to-mesenchymal transition in the development of endometriosis. Oncotarget.

[26] Ge S.X., Jung D., Yao R. (2020). ShinyGO: A graphical gene-set enrichment tool for animals and plants. Bioinformatics.

[27] Subramanian A., Tamayo P., Mootha V.K., Mukherjee S., Ebert B.L., Gillette M.A., Paulovich A., Pomeroy S.L., Golub T.R., Lander E.S. (2005). Gene set enrichment analysis: A knowledge-based approach for interpreting genome-wide expression profiles. Proc. Natl. Acad. Sci. USA.

[28] Mootha V.K., Lindgren C.M., Eriksson K.F., Subramanian A., Sihag S., Lehar J., Puigserver P., Carlsson E., Ridderstrale M., Laurila E. (2003). PGC-1alpha-responsive genes involved in oxidative phosphorylation are coordinately downregulated in human diabetes. Nat. Genet..

[29] Toshima J., Toshima J.Y., Amano T., Yang N., Narumiya S., Mizuno K. (2001). Cofilin phosphorylation by protein kinase testicular protein kinase 1 and its role in integrin-mediated actin reorganization and focal adhesion formation. Mol. Biol. Cell.

[30] Klemmt P.A.B., Starzinski-Powitz A. (2018). Molecular and Cellular Pathogenesis of Endometriosis. Curr. Womens Health Rev..

[31] Zang C., Nie F.Q., Wang Q., Sun M., Li W., He J., Zhang M., Lu K.H. (2016). Long non-coding RNA LINC01133 represses KLF2, P21 and E-cadherin transcription through binding with EZH2, LSD1 in non small cell lung cancer. Oncotarget.

[32] Liu Y., Tang T., Yang X., Qin P., Wang P., Zhang H., Bai M., Wu R., Li F. (2021). Tumor-derived exosomal long noncoding RNA LINC01133, regulated by Periostin, contributes to pancreatic ductal adenocarcinoma epithelial-mesenchymal transition through the Wnt/beta-catenin pathway by silencing AXIN2. Oncogene.

[33] Hanieh H., Ahmed E.A., Vishnubalaji R., Alajez N.M. (2020). SOX4: Epigenetic regulation and role in tumorigenesis. Semin. Cancer Biol..

[34] Tiwari N., Tiwari V.K., Waldmeier L., Balwierz P.J., Arnold P., Pachkov M., Meyer-Schaller N., Schubeler D., van Nimwegen E., Christofori G. (2013). Sox4 is a master regulator of epithelial-mesenchymal transition by controlling Ezh2 expression and epigenetic reprogramming. Cancer Cell.

[35] Hazan R.B., Qiao R., Keren R., Badano I., Suyama K. (2004). Cadherin switch in tumor progression. Ann. N. Y. Acad. Sci..

[36] Thiery J.P., Acloque H., Huang R.Y., Nieto M.A. (2009). Epithelial-mesenchymal transitions in development and disease. Cell.

[37] Wheelock M.J., Jensen P.J. (1992). Regulation of keratinocyte intercellular junction organization and epidermal morphogenesis by E-cadherin. J. Cell Biol..

[38] Matsuzaki S., Darcha C. (2012). Epithelial to mesenchymal transition-like and mesenchymal to epithelial transition-like processes might be involved in the pathogenesis of pelvic endometriosis. Hum. Reprod..

[39] Van Patten K., Parkash V., Jain D. (2010). Cadherin expression in gastrointestinal tract endometriosis: Possible role in deep tissue invasion and development of malignancy. Mod. Pathol..

[40] Kuessel L., Wenzl R., Proestling K., Balendran S., Pateisky P., Yotova S., Yerlikaya G., Streubel B., Husslein H. (2017). Soluble VCAM-1/soluble ICAM-1 ratio is a promising biomarker for diagnosing endometriosis. Hum. Reprod..

[41] Zhang J., Li H., Yi D., Lai C., Wang H., Zou W., Cao B. (2019). Knockdown of vascular cell adhesion molecule 1 impedes transforming growth factor beta 1-mediated proliferation, migration, and invasion of endometriotic cyst stromal cells. Reprod. Biol. Endocrinol..

[42] Wu T.C. (2007). The role of vascular cell adhesion molecule-1 in tumor immune evasion. Cancer Res..

[43] Schlesinger M., Bendas G. (2015). Vascular cell adhesion molecule-1 (VCAM-1)--an increasing insight into its role in tumorigenicity and metastasis. Int. J. Cancer.

[44] Yotova I., Quan P., Gaba A., Leditznig N., Pateisky P., Kurz C., Tschugguel W. (2012). Raf-1 levels determine the migration rate of primary endometrial stromal cells of patients with endometriosis. J. Cell. Mol. Med..

[45] Morris K., Ihnatovych I., Ionetz E., Reed J., Braundmeier A., Strakova Z. (2011). Cofilin and slingshot localization in the epithelium of uterine endometrium changes during the menstrual cycle and in endometriosis. Reprod. Sci..

[46] Yuge A., Nasu K., Matsumoto H., Nishida M., Narahara H. (2007). Collagen gel contractility is enhanced in human endometriotic stromal cells: A possible mechanism underlying the pathogenesis of endometriosis-associated fibrosis. Hum. Reprod..

[47] Toshima J., Ohashi K., Okano I., Nunoue K., Kishioka M., Kuma K., Miyata T., Hirai M., Baba T., Mizuno K. (1995). Identification and characterization of a novel protein kinase, TESK1, specifically expressed in testicular germ cells. J. Biol. Chem..

[48] Bamburg J.R., McGough A., Ono S. (1999). Putting a new twist on actin: ADF/cofilins modulate actin dynamics. Trends Cell Biol..

[49] Sander E.E., ten Klooster J.P., van Delft S., van der Kammen R.A., Collard J.G. (1999). Rac downregulates Rho activity: Reciprocal balance between both GTPases determines cellular morphology and migratory behavior. J. Cell. Biol..

[50] Fassbender A., Rahmioglu N., Vitonis A.F., Vigano P., Giudice L.C., D’Hooghe T.M., Hummelshoj L., Adamson G.D., Becker C.M., Missmer S.A. (2014). World Endometriosis Research Foundation Endometriosis Phenome and Biobanking Harmonisation Project: IV. Tissue collection, processing, and storage in endometriosis research. Fertil. Steril..

[51] Zeitvogel A., Baumann R., Starzinski-Powitz A. (2001). Identification of an invasive, N-cadherin-expressing epithelial cell type in endometriosis using a new cell culture model. Am. J. Pathol..

[52] Banu S.K., Lee J., Starzinski-Powitz A., Arosh J.A. (2008). Gene expression profiles and functional characterization of human immortalized endometriotic epithelial and stromal cells. Fertil. Steril..

[53] Dobin A., Davis C.A., Schlesinger F., Drenkow J., Zaleski C., Jha S., Batut P., Chaisson M., Gingeras T.R. (2013). STAR: Ultrafast universal RNA-seq aligner. Bioinformatics.

[54] R Core Team (2020). R: A Language and Environment for Statistical Computing.

[55] Love M.I., Huber W., Anders S. (2014). Moderated estimation of fold change and dispersion for RNA-seq data with DESeq2. Genome Biol..

[56] Li H., Handsaker B., Wysoker A., Fennell T., Ruan J., Homer N., Marth G., Abecasis G., Durbin R. (2009). The Sequence Alignment/Map format and SAMtools. Bioinformatics.

[57] Trapnell C., Roberts A., Goff L., Pertea G., Kim D., Kelley D.R., Pimentel H., Salzberg S.L., Rinn J.L., Pachter L. (2012). Differential gene and transcript expression analysis of RNA-seq experiments with TopHat and Cufflinks. Nat. Protoc..

[58] Livak K.J., Schmittgen T.D. (2001). Analysis of relative gene expression data using real-time quantitative PCR and the 2(-Delta Delta C(T)) Method. Methods.

[59] Rubiolo C., Piazzolla D., Meissl K., Beug H., Huber J.C., Kolbus A., Baccarini M. (2006). A balance between Raf-1 and Fas expression sets the pace of erythroid differentiation. Blood.

[60] Yotova I., Hsu E., Do C., Gaba A., Sczabolcs M., Dekan S., Kenner L., Wenzl R., Tycko B. (2017). Epigenetic alterations affecting transcription factors and signaling pathways in stromal cells of endometriosis. PLoS ONE.

[61] Hammond L., Fitzpatrick M. (2020). Measuring cell fluoresecence using ImageJ. The Open Lab Book, Release 1.0.

