# Supplementary material for: LINC01133 Inhibits Invasion and Promotes Proliferation in an Endometriosis Epithelial Cell Line
Source: Int J Mol Sci. 2021 Aug 4;22(16):8385. doi: 10.3390/ijms22168385 (PMC8395043; doi:10.3390/ijms22168385)
Supplement: Supplementary file 1 [file ijms-22-08385-s001.zip › ijms-1290378-supplementary figures.pdf]

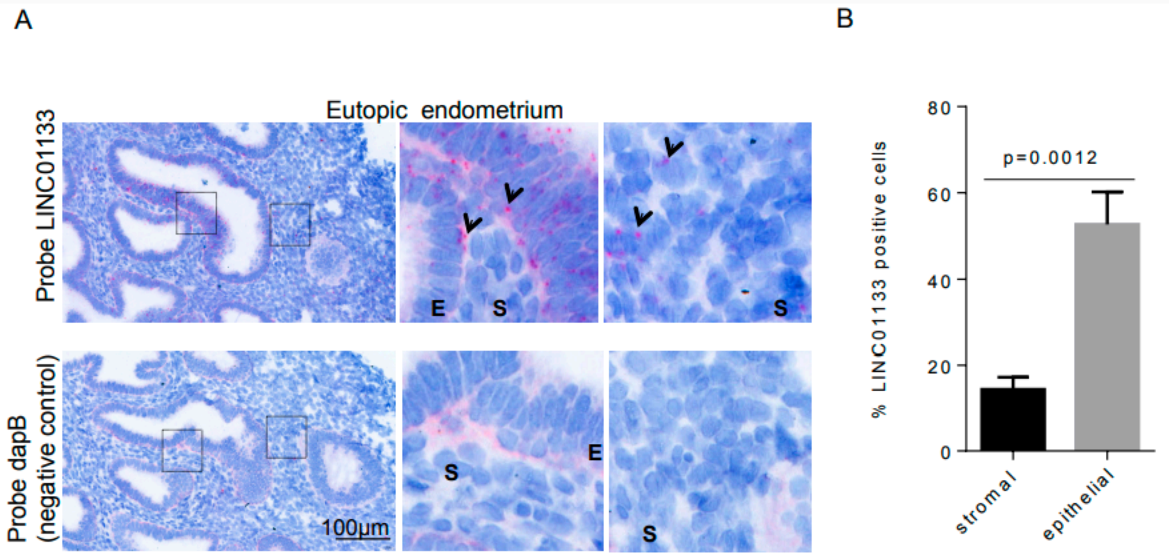

Figure S1. LINC01133 is expressed in both the epithelial and stroma cell compartments of endometriosis tissue. (**A**) Representative RNAscope® in situ hybridization slide scan images show expression of LINC01133 in the eutopic endometrium of an endometriosis patient (top), while expression is absent in the dapB negative control (bottom). LINC01133 specific probe (red); negative control probe DapB (red); hematoxylin staining (blue) was used to visualize the cell nuclei on paraffin slides. Magnification scale of 100 µm is on the figure. An example for positive signal for LINC01133 is indicated with a black arrow. (**B**) LINC01133 expression is higher in the epithelial than in the stroma cell compartment in eutopic endometrial tissue samples of women with endometriosis. The number of positive cells in each tissue compartment was counted using the ImageJ program from n=3 independent samples. The data is plotted as percent positive cells relative to the total number of cells in each slide for each tissue compartment. Statistical analysis of the data was performed by Man-Whitney test. The p-value is indicated on the top of the graph.

A

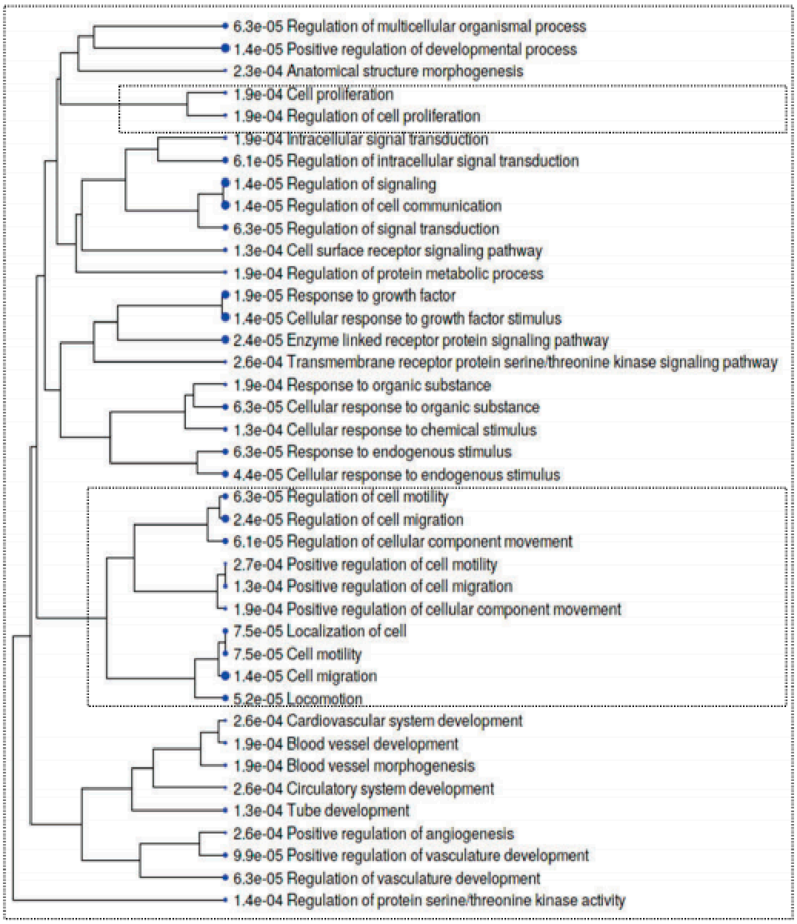

B

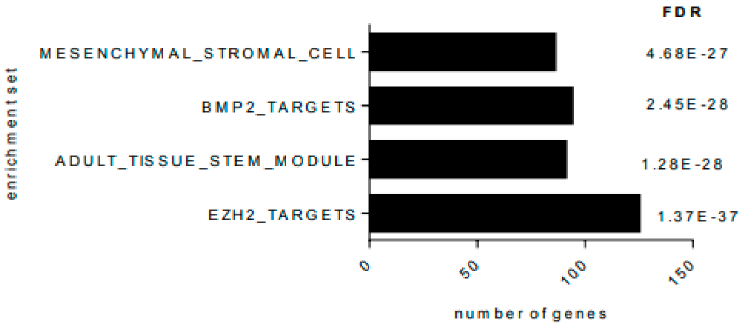

Figure S2. Gene-ontology (GO) and gene-set (GS) enrichment analysis for DE genes between LINC01133 knockdown and control siRNA transfected 12Z cells. (**A**) A hierarchical clustering three summarizing the correlation among significant gene ontology terms after GO analysis of the list of DE genes under LICO1133 knockdown is shown. The biological pathways with many shared genes are clustered together and bigger dots indicate more significant p-value. The most relevant pathways for pathogenesis of endometriosis are marked with black dotted lines. p-value cut-off < 0.05 was used. (**B**) Graphical representation of top enriched gene sets identified by SG enrichment analysis of the list of DE genes between LIC01133 knockdown and control siRNA transfected cells 12Z cells. FDR for each set is given on the right side of the graph the number of gene in each set is plotted on x-axis and the name of the set on y-axis. A cut-off with FDR < 0.05 was used.

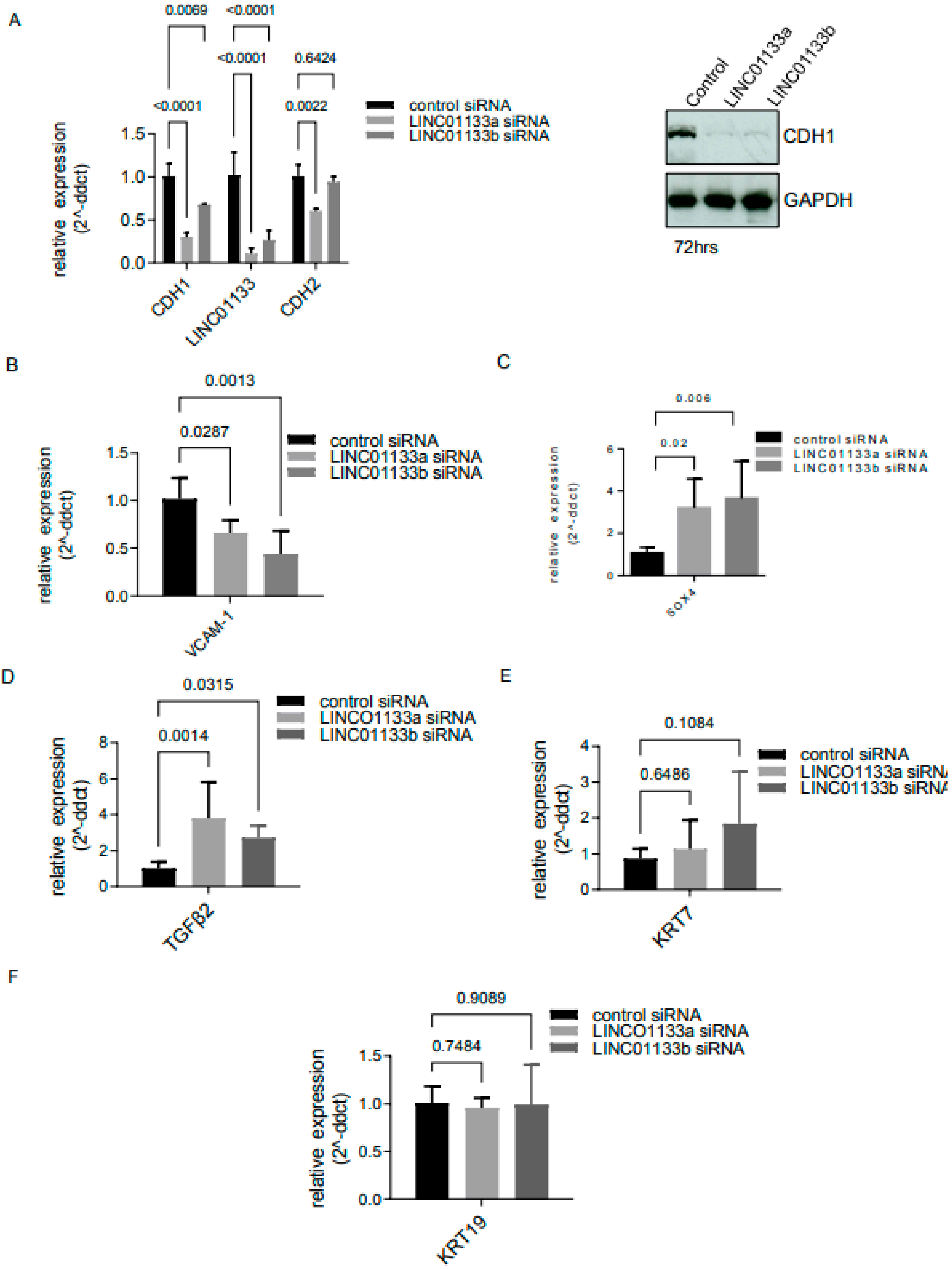

Figure S3. Validation of RNA-sequencing by Q-PCR of genes with known or suggested functions in EMT The figure shows average gene expression levels of selected genes and average protein levels of CDH1 in 12Z cells from six and respectively, three biological replicates, 72 hrs after LINC01133 knockdown. All expression values were normalized to the mean of the control siRNA treated cells set to 1. (**A**) LINC01133 knockdown leads to a significant decrease in CDH1 for independent siRNA oligos, while only the most efficient LINC01133 knockdown leads to a significant decrease in CDH2

levels by RT-qPCR (Left). A reduction in CDH1 protein levels is also seen following LINC01133 knockdown with independent siRNA oligos (Right). (**B**) The relative expression levels of VCAM-1 are decreased in LINC01133 knockdown cells. (**C**) LINC01133 knockdown significantly increased the levels of expression of SOX4 gene. (**D**) Upregulation of the relative levels of expression of TGFβ2 gene in LINC01133 knockdown cells is shown. The effects of the knockdown on gene expression are dependent on LINC01133 knockdown efficiency. (**E**) LINC01133 knockdown does not significantly affect KRT7 expression. (**F**) LINC01133 knockdown does not significantly affect KRT19 expression. Statistical analysis of the data is performed by ordinary one way ANOVA, followed by Dunnett's multiple comparison test. Statistical analysis of the data is performed by ordinary one way ANOVA, followed by Dunnett's multiple comparison test. Adjp < 0.05 was considered significant.

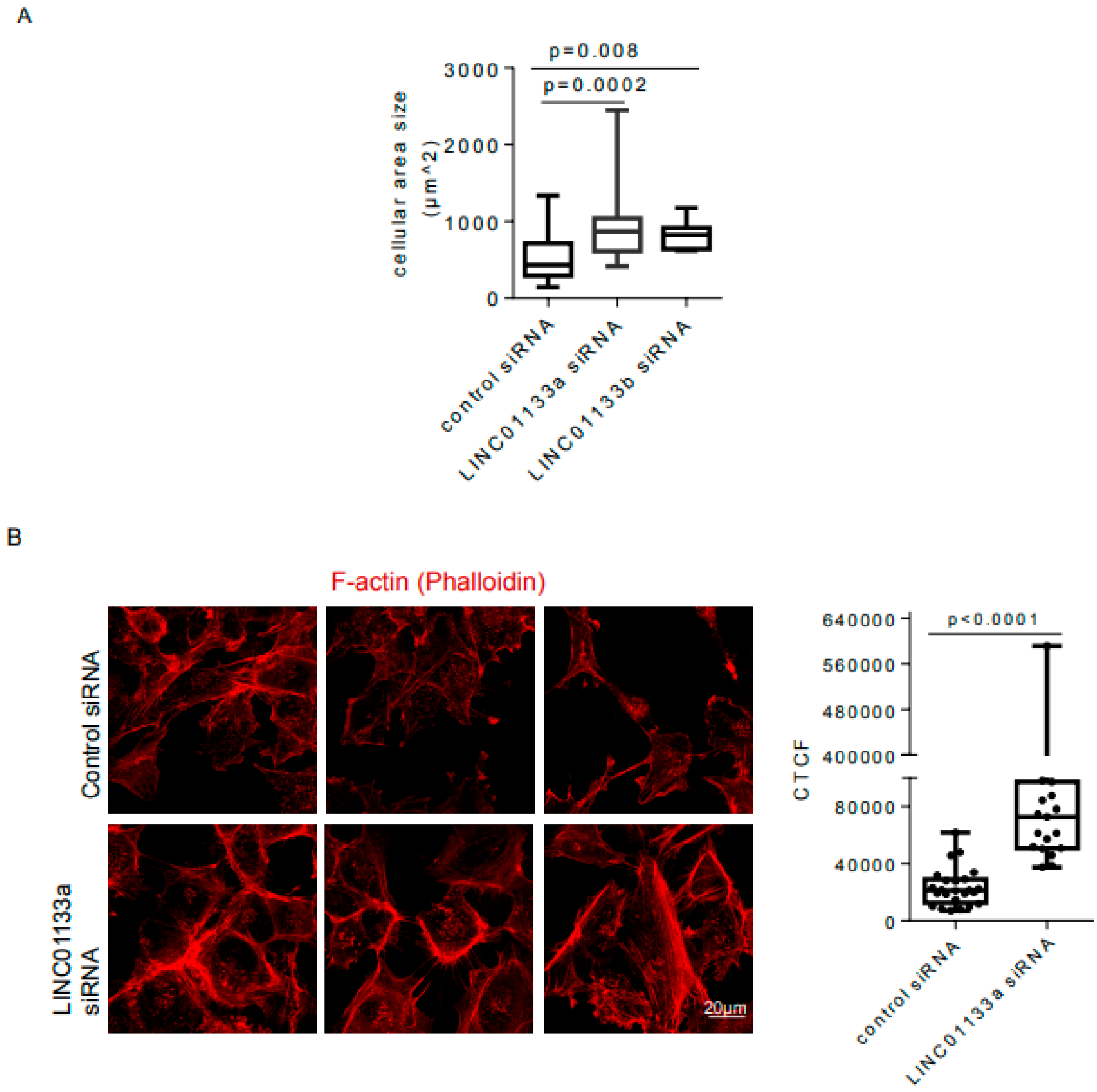

Figure S4. LINC01133 knockdown effects the cross-sectional cell area and the density of F-Actin stress fibers. (**A**) LINC01133 knockdown increases the cross-sectional cell area (µm2) of 12Z endometriosis epithelial cells. F-actin (Phalloidin) immunofluorescence was conducted on 8- well chamber slides for control, LINC0113a and LINC01133b siRNA knockdowns 72 hours after transfection. For each treatment, cross-sectional cell area was calculated from four independent images using ImageJ as described in the methods (x63 Leica Sp8 Confocal). Data is presented as a box plot ranging from minimum to maximum, including the median and box boundaries at the 25th and 75th percentiles. Statistically significant differences between the groups are indicated on the top of each panel (adjp <0.05 Kruskal-Wallis test with Dunn's for multiple comparison). (**B**) Left: Immunofluorescence of F-actin (Phalloidin) 72 hrs after transfection with siRNA control and the LINC01133a oligo shows an increase in the number and/or density of Actin stress fibers. Representative confocal images are shown. Right: F-actin (Phalloidin) immunofluorescence is significantly increased following LINC01133 knockdown, indicating that F-actin stress fibers are increased. Corrected total single cell Phalloidin fluorescence (CTCF) was calculated for control and LINC01133a siRNA knockdown using ImageJ from three independent images shown in (**A**), as described in the methods. The data is

presented as a boxplot with individual cell CTCF levels plotted as black dots, and the median and box boundaries at the 25th and 75th percentiles. Statistically significant differences between the groups are indicated on the top of the graph (Mann-Whitney test, adjp <0.0001)
